# Clonal Diversity, Biofilm Formation, and Antimicrobial Resistance among *Stenotrophomonas maltophilia* Strains from Cystic Fibrosis and Non-Cystic Fibrosis Patients

**DOI:** 10.3390/antibiotics9010015

**Published:** 2020-01-02

**Authors:** Arianna Pompilio, Vincenzo Savini, Ersilia Fiscarelli, Giovanni Gherardi, Giovanni Di Bonaventura

**Affiliations:** 1 Department of Medical, Oral and Biotechnological Sciences, and Center of Advanced Sciences and Technology (CAST), “G. d’Annunzio” University of Chieti-Pescara, Via Luigi Polacchi 11, 66100 Chieti, Italy; arianna.pompilio@unich.it; 2 Clinical Microbiology and Virology, Spirito Santo Hospital, Via Fonte Romana 8, 65124 Pescara, Italy; vincenzosavini@libero.it; 3 Laboratory of Cystic Fibrosis Microbiology, “Bambino Gesú” Hospital, Piazza di Sant’Onofrio 4, 00165 Roma, Italy; evita.fiscarelli@opbg.net; 4 Campus Biomedico University of Rome, Via Álvaro del Portillo 21, 00128 Roma, Italy; G.Gherardi@unicampus.it

**Keywords:** *Stenotrophomonas maltophilia*, biofilm formation, antibiotic resistance

## Abstract

The intrinsic antibiotic resistance of *Stenotrophomonas maltophilia*, along with its ability to form biofilm both on abiotic surfaces and host tissues, dramatically affects the efficacy of the antibiotic therapy. In this work, 85 *S. maltophilia* strains isolated in several hospital of central Italy and from several clinical settings were evaluated for their genetic relatedness (by pulsed-field gel electrophoresis, PFGE), biofilm formation (by microtiter plate assay), and planktonic antibiotic resistance (by Kirby–Bauer disk diffusion technique). The *S. maltophilia* population showed a high genetic heterogeneity: 64 different PFGE types were identified, equally distributed in cystic fibrosis (CF) and non-CF strains, and some consisted of multiple strains. Most of the strains (88.2%) were able to form biofilm, although non-CF strains were significantly more efficient than CF strains. CF strains produced lower biofilm amounts than non-CF strains, both those from respiratory tracts and blood. Non-CF PFGE types 3 and 27 consisted of strong-producers only. Cotrimoxazole and levofloxacin were the most effective antibiotics, being active respectively against 81.2% and 72.9% of strains. CF strains were significantly more resistant to piperacillin/tazobactam compared to non-CF strains (90% versus 53.3%), regardless of sample type. Among respiratory strains, cotrimoxazole was more active against non-CF than CF strains (susceptibility rates: 86.7% versus 75%). The multidrug resistant phenotype was significantly more prevalent in CF than non-CF strains (90% versus 66.7%). Overall, the multidrug-resistance level was negatively associated with efficiency in biofilm formation. Our results showed, for the first time, that in *S. maltophilia* both classical planktonic drug resistance and the ability of biofilm formation might favor its dissemination in the hospital setting. Biofilm formation might in fact act as a survival mechanism for susceptible bacteria, suggesting that clinical isolates should be routinely assayed for biofilm formation in diagnostic laboratories.

## 1. Introduction

Among the “emerging” pathogens recognized in the recent years, *Stenotrophomonas maltophilia* plays a significant role in colonization and infection in hospital, and less often, community settings. This opportunistic pathogen has, in fact, been implicated in a variety of nosocomial infections, especially in intensive care unit patients (such as ventilator-associated pneumonia and sepsis), life-threatening diseases in immunocompromised patients with hematological malignancies and cancers, and respiratory tract infections in patients with chronic lung diseases [1,2].

*S. maltophilia* commonly causes pneumonia, bacteremia, sepsis, and wound infections, and less commonly, urinary tract infections, endocarditis, soft tissue infections, meningitis, osteochondritis, peritonitis, and ophthalmic infections [1,2]. Although it is not considered a highly virulent pathogen, *S. maltophilia* has been associated with high crude mortality, ranging from 25% to 75% in the case of pneumonia and from 14% to 69% in the case of bacteremia [2].

Although *Pseudomonas aeruginosa* is the most prevalent pathogen in cystic fibrosis (CF) patients, *S. maltophilia* is being increasingly isolated from CF airways, due to its ability to evade many antipseudomonal antibiotics [3,4,5,6]. In this clinical setting, the microorganism can account for perseverant colonization and chronic infection, although the clinical relevance in these patients is yet unclear. In fact, despite some studies have defined this microorganism as a colonizer, others demonstrated that its presence should not be ignored in some CF patients as *S. maltophilia* is associated with an increased risk of pulmonary exacerbations, the deterioration in pulmonary function, the need for lung transplantation, and death [3,4,5,6].

The biofilm-forming ability of *S. maltophilia* has increasingly been accepted as an important virulence trait. This microorganism can form biofilms both on abiotic surfaces and host tissues, dramatically enhancing the resistance to therapeutically important antibiotics, including aminoglycosides, fluoroquinolones, and tetracycline [7,8,9,10]. Therefore, biofilm formation might play a relevant role in the persistence of *S. maltophilia* infection in hospital settings, especially in CF patients where it complicates the therapeutic management of bronchial colonization- and infection [11,12,13].

However, biofilm formation is not the only reason for antimicrobial treatment failure. In fact, a distinctive feature of *S. maltophilia* strains is their resistance to a wide range of antibiotics, which makes these infections difficult to treat [14,15]. Nonetheless, contrarily to *P. aeruginosa*, treatment protocols are not yet standardized for *S. maltophilia*, and limited data are available about susceptibility profiles, highlighting the importance for studying the susceptibility patterns of this microorganism.

The connection between biofilm formation and planktonic antibiotic resistance is of considerable interest to biomedical researchers, although studies performed in this regard over the past two decades have yielded conflicting results, and therefore, inconsistent information [16,17,18,19,20,21]. Particularly, although some studies found a positive relationship between biofilm formation and antibiotic resistance in *Staphylococcus aureus* [16], *Acinetobacter baumannii* [18], and *Staphylococcus epidermidis* [19], in others an opposite trend was observed for *A. baumannii* [20,21].

To the best of our knowledge, a rigorous investigation in this regard has not been carried out for *S. maltophilia*. Therefore, in this work, we assessed the antimicrobial resistance of planktonic cells, the efficiency of biofilm formation, and the clonal relatedness in *S. maltophilia*. Such evaluations were extended to a larger collection of strains isolated from several clinical samples, both from CF and non-CF patients, to evaluate a possible relationship among biofilm formation ability, clinical source, and antibiotic resistance pattern. With this aim, 85 *S. maltophilia* strains were evaluated for their genetic relatedness by pulsed-field gel electrophoresis, antibiotic resistance by Kirby–Bauer disk diffusion technique, and capability for biofilm development in 96-well microtiter plates.

Overall, our findings highlighted that: (i) *S. maltophilia* enhances its persistence—both environmental and into the host—by a smart balance between antibiotic resistance and biofilm formation; and (ii) biofilm formation ability should be evaluated along with antimicrobial susceptibility testing to improve the efficacy of the treatment against biofilm-related infections.

## 2. Results

### 2.1. The S. maltophilia Population Shows a High Genetic Heterogeneity

The genetic diversity and the clonal relatedness of the tested strains were assessed by PFGE analysis, and results are shown in Table 1.

A total of 64 different PFGE types were identified among the 85 *S. maltophilia* strains, with 30 and 34 different PFGE profiles observed among 40 CF and 45 non-CF strains, respectively. These findings indicated that the genetic heterogeneity—calculated as (number of pulsotypes/number of strains tested) x 100—is comparable in both CF and non-CF strains (75% versus 75.5%, respectively).

Six PFGE types—specifically two and four, respectively, among CF and non-CF strains—were represented by multiple strains. Among CF strains, PFGE type 9 consisted of 10 strains, followed by PFGE type 50, comprised of two strains. Among non-CF strains, PFGE type 3 was the most prevalent (six strains), followed by PFGE type 27 (five strains), and PFGE types 21 and 40 (two strains each). No PFGE types shared by CF and non-CF strains were found.

### 2.2. Antibiotic Resistance Is More Prevalent in CF Strains

**Figure 1:**
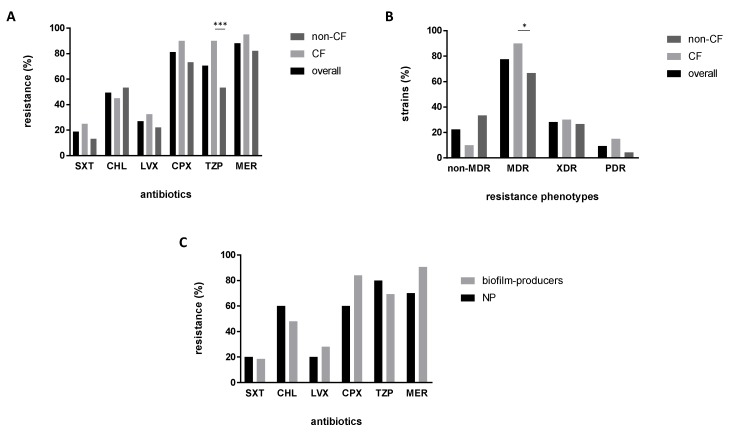
Distribution of antibiotic resistance rates and profiles. *S. maltophilia* strains were evaluated for in vitro susceptibility to six antibiotics: SXT, cotrimoxazole; CHL, chloramphenicol; LVX, levofloxacin; CPX, ciprofloxacin; TZP, piperacillin/tazobactam; MER, meropenem. The frequencies of (**A**) antibiotic resistances, and (**B**) multiresistance profiles (non-MDR, MDR, XDR, and PDR) were calculated for cystic fibrosis (CF) (*n* = 40), non-CF (*n* = 45), and overall (*n* = 85) strains. (**C**) Resistance rates were calculated according to the capability to form biofilm: Non-producers (NP; *n* = 10) or biofilm-producers (*n* = 75; gathering strong, moderate, and weak-producers). Significance level at Fisher’s exact test: * *p* < 0.05; *** *p* < 0.001. Resistance profiles according to Magiorakos et al. [24]: MDR, multidrug-resistant strains; XDR, extensively drug-resistant strains; PDR, pandrug-resistant strains.

The in vitro activity of six antibiotics against *S. maltophilia* strains was measured by disk diffusion agar or broth microdilution, and results are shown in detail in Table 1 and summarized in Figure 1. Considering the strains as a whole, cotrimoxazole and levofloxacin were the most effective antibiotics, being active respectively against 81.2% and 72.9% of strains. Contrarily, the higher resistance rates were observed for meropenem (88.2%), followed by ciprofloxacin (81.2%), piperacillin/tazobactam (70.6%), and chloramphenicol (49.4%) (Figure 1A).

The strains isolated from CF airways exhibited significantly higher resistance rates to piperacillin/tazobactam compared to those isolated from non-CF patients (90% versus 53.3%, respectively; *p* < 0.001) (Figure 1A); the same trend was confirmed after stratification based on sample type (90% versus 62% and 36.4%, respectively, for respiratory from CF patients, respiratory from non-CF patients, and blood from non-CF patients; *p* < 0.01) (data not shown). Cotrimoxazole showed comparable activity in both CF and non-CF strains; however, when only respiratory strains were considered, the antibiotic was significantly more active against non-CF than CF strains (susceptibility rates: 86.7% versus 75%, respectively; *p* < 0.05) (Figure 1A).

The antibiotic resistance patterns revealed that most strains (66 out of 85, 77.6%) show multidrug resistance, with extensively drug-resistant (XDR) and pandrug-resistant (PDR) phenotypes, respectively, seen for 28.2% and 9.4% of the strains.

The multidrug-resistant (MDR) phenotype was observed in a proportion significantly higher in CF strains compared with non-CF strains (90% versus 66.7%, respectively; *p* < 0.05) (Figure 1B). CF strains also showed higher XDR and PDR prevalence compared with non-CF strains, although not at a statistically significant extent (XDR: 30% versus 26.7%, respectively; PDR: 15% versus 4.4%, respectively).

No relationship between specific PFGE types and antibiotic resistance was found.

### 2.3. Non-CF Strains Are Significantly More Efficient Than CF Strains in Forming Biofilm

**Figure 2:**
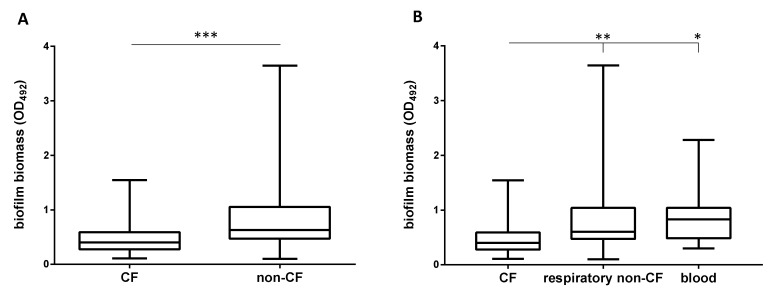
Biofilm formation according to patient and sample types. Biofilm biomass, assessed by spectrophotometric assay after crystal violet assay, was stratified according to (**A**) patients with (CF; *n* = 40) or without (non-CF; *n* = 45) cystic fibrosis, and (**B**) sample type (CF, *n* = 40; respiratory non-CF, *n* = 29; blood, *n* = 11). Results are shown as box and whiskers: the ends of the whiskers represent the minima and the maxima of all the data; the box always extends from the 25th to 75th percentiles, while the line in the middle of the box is plotted at the median. Significance level from Mann–Whitney test: * *p* < 0.05; ** *p* < 0.01; *** *p* < 0.001.

**Figure 3:**
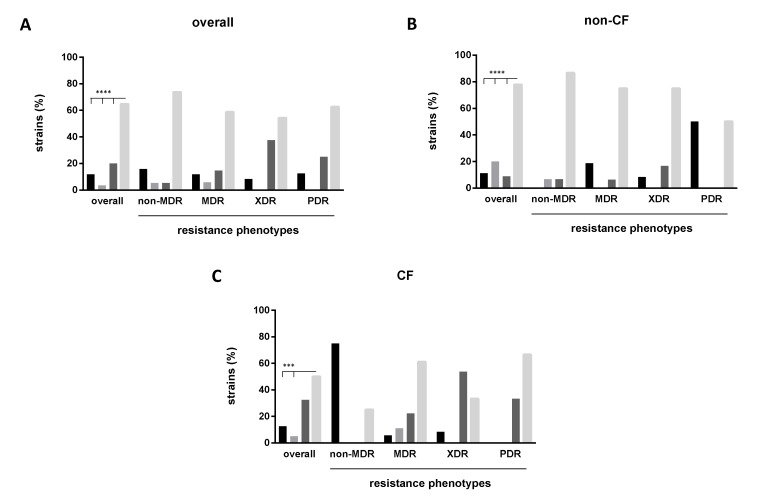
Biofilm formation and multidrug-resistant phenotypes. Biofilm formation was assessed by spectrophotometric assay after crystal violet assay and categorized according to Stepanovic et al. [23]: Non-producers, weak-producers, moderate-producers, and strong-producers (left-to-right, in each series of histograms). Susceptibility tests were performed using Kirby-Bauer disk diffusion agar. Resistance profiles according to Magiorakos et al. [24]: MDR, multidrug-resistant strains; XDR, extensively drug-resistant strains; PDR, pandrug-resistant strains. Significance level at Fisher’s exact test: *** *p* < 0.001; **** *p* < 0.0001.

The biofilm forming ability was assessed on polystyrene using the microtiter plate method, and results are shown in detail in Table 1 and summarized in Figure 2 and Figure 3. Most of the strains (75 out of 85, 88.2%) were able to form biofilms on polystyrene, although to different extents, as indicated by OD_492_ values (range: 0.100–3.646; mean ± SD: 0.745 ± 0.644; coefficient of variation: 86.4%) (Table 1). The prevalence of strains able to form biofilm was comparable both in CF and non-CF strains (87.5 versus 88.9%, respectively), although the median amount of biofilm formed by non-CF strains was significantly higher than that produced by CF strains (OD_492_, median: 0.631 versus 0.400, respectively; *p* < 0.001) (Figure 2A). Biofilm formation was related to the source of isolation and patient type since CF strains produced lower biofilm biomass levels compared to those isolated from non-CF patients both from airways (OD_492_, median: 0.400 versus 0.604, respectively; *p* < 0.01) and blood (OD_492_, median: 0.400 versus 0.831, respectively; *p* < 0.05), whereas no significant differences were found among non-CF strains regardless of the sample type considered (Figure 2B).

Categorization of biofilm formation according to Stepanovic et al. [23] showed that, overall, strong-producers were significantly more prevalent compared with other biofilm classes (64.7% versus 20%, 3.5%, and 11.8%, respectively for strong, moderate, weak, and non-producers; *p* < 0.0001) (Figure 3A). The same trend was observed both among non-CF (77.8%; *p* < 0.0001 versus other classes) (Figure 3B) and CF (50%; *p* < 0.0003 versus other classes except for moderate-producers) strains (Figure 3C). Further, among CF strains, the proportion of moderate-producers was higher compared to that observed for weak-producers (32.5 versus 2.5%, respectively; *p* < 0.01). The frequency of strong-producer strains was higher among the non-CF group than CF one (77.8% versus 50%, respectively; *p* < 0.05). Particularly, most of the strains from blood were strong-producers (six out of nine, 66.6%), suggesting that biofilm formation might play a role in *S. maltophilia*‘s tissue invasion.

Non-CF PFGE types 3 (*n* = six strains) and 27 (*n* = five strains) consisted of strong-producers only, whereas CF PFGE type 9 consisted mostly of strong-producers (six out of 10, 60%), along with moderate (three out of 10, 30%) and weak-producers (one out of 10, 10%) strains (Table 1).

### 2.4. Biofilm Formation and Antibiotic Resistance Are Inversely Related

**Figure 4:**
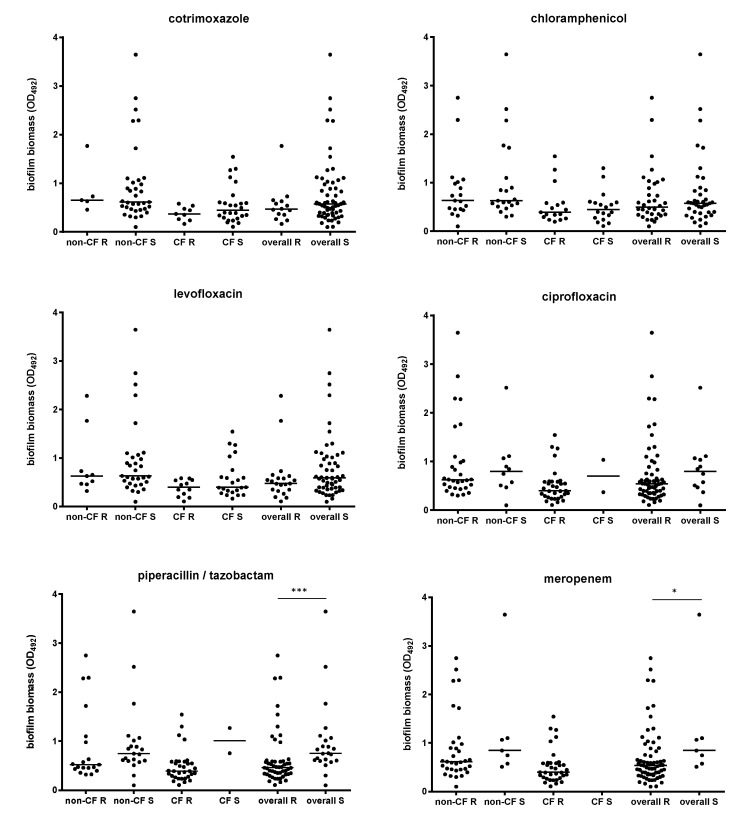
Biofilm formation according to antibiotic resistance and patient type. Biofilm biomass formation, spectrophotometrically assessed by crystal violet assay, was stratified on each antibiotic tested—according to susceptibility (S) or resistance (R)—and patient type. Results are shown as scatter plots, with horizontal lines indicating the median values. Significance level at Mann–Whitney test: * *p* < 0.05; *** *p* < 0.0001.

Trying to find a relationship between biofilm formation and planktonic resistance to antibiotics, we evaluated the variations in the biofilm biomass formed on the bases of both susceptibility/resistance and multi-resistance profiles. Considering the strains as a whole, those susceptible to piperacillin/tazobactam or meropenem produced significantly more biofilm than resistant counterparts (OD_492_, median; piperacillin/tazobactam: 0.755 versus 0.464, *p* < 0.001; meropenem: 0.847 versus 0.536, *p* < 0.05; respectively for susceptible and resistant strains) (Figure 4).

**Figure 5:**
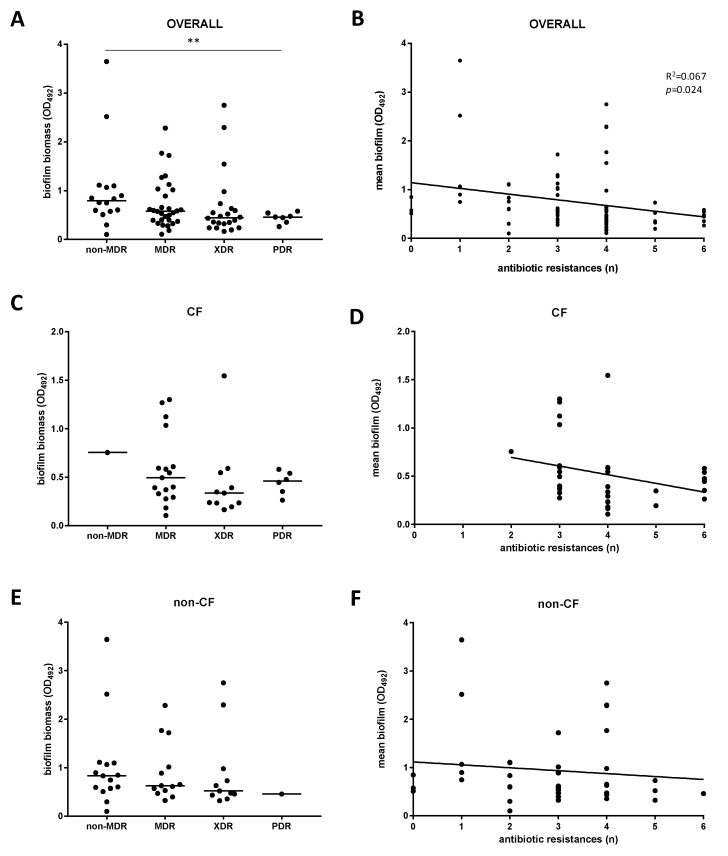
Biofilm formation according to the antibiotic resistance level. Biofilm formation was measured by crystal violet assay, and antibiotic resistance by disk diffusion technique. Biofilm formation was stratified according to (**A**,**C**,**E**) the resistance phenotype (non-MDR, MDR, XDR, PDR)— showing results as scatter plot, with the horizontal line indicating the median value—and (**B**,**D**,**F**) the number of resistances to each antibiotic tested. Significance level: ** *p* < 0.01, Mann-–Whitney test; r^2^: 0.067, *p* = 0.024, linear regression analysis.

Next, stratifying the biofilm biomass formation on the resistance phenotype, we found that non-MDR strains were more efficient in forming biofilm compared to PDR strains (OD_492_, median: 0.793 versus 0.459, respectively; *p* < 0.01) (Figure 5A), whereas no significant differences were observed among CF (Figure 5C) and non-CF (Figure 5E) strains. The multidrug-resistance level—calculated as the number of antibiotic resistances showed by each strain—was negatively associated with mean biofilm formed only when strains were considered as a whole (*p* = 0.024, linear regression analysis) (Figure 5B). A similar, although not statistically significant, trend was observed among both CF (Figure 5D) and non-CF (Figure 5F) strains. Confirming this trend, the frequency of non-producer strains observed for non-MDR group was significantly higher compared with other groups considered (75% versus 5.6%, 8.3%, and 0%, respectively, for MDR, XDR, and PDR strains; *p* < 0.01) (Figure 3C). Further, both in “overall” and non-CF groups, the frequency of strong-producers was higher in non-MDR group compared with MDR, XDR, and PDR, although these differences were not statistically significant (Figure 3A,B).

The resistance rates exhibited by biofilm non-producer strains were comparable to those observed among biofilm-producers (weak, moderate, and strong) (Figure 1C). Non-producer strains showed both MDR (seven out of 10; 70%) and non-MDR (three out of 10; 30%) phenotypes.

## 3. Discussion

The present study was undertaken to investigate the clonal relatedness, antibiotic-resistance patterns, and biofilm forming ability of a population of *S. maltophilia* strains isolated from different hospitals in central Italy, as a representative for several clinical settings. The possible relationship among these traits was also assessed.

The persistence of bacterial infections is due to the emergence of persister cells, whose physiologically dormant state makes them able to elude antibiotic killing. The selection for persister cells occurs under conditions that include hostile host environments, a damage response being caused by sublethal concentrations of antibiotics, and bacterial biofilms [25].

Biofilms are microbial communities wrapped in a polysaccharidic matrix produced by the bacteria, which are adhered to an inert or biotic surface. Biofilms are far less susceptible to antibiotics than planktonic cells, and therefore, they have been associated with a wide range of infections in a clinical setting, from those related to exogenous devices (i.e., catheters or prosthetic joints) to chronic tissue infections, such as those occurring in the lungs of CF patients [26,27].

In this work, we found that biofilm forming ability is highly preserved in *S. maltophilia* and could, therefore, be accepted as an important virulence trait underlying treatment failure, recurrences, and/or persistence of colonization. Biofilm formation appeared to be related to both the site of infection and patient type. In fact, respiratory strains from CF lung were less efficient in forming biofilm with respect to those isolated from the airways of patients without CF, as indicated by differences in both median biofilm biomass and frequency of strong-producers, suggesting that *S. maltophilia* can successfully adapt to a highly stressful environment, such as a CF lung, by paying a “biological cost.”

Molecular analysis of *XbaI*-digested DNA resulted in 64 distinct PFGE types; 90.6% of those consisted of only one isolate, confirming the great flexibility of *S. maltophilia* to evolve, regardless of the clinical setting considered [11,28,29]. On the other hand, the evidence for PFGE types consisting of multiple strains shows that the same strain was isolated from different patients over a period, emphasizing the persistence and dissemination of *S. maltophilia* in the hospital environment. This extraordinary variability, besides suggesting that most strains were acquired independently rather than because of cross-transmission, could also be the consequence of the high propensity to form biofilm we found in this study. In fact, earlier observations showed that the characteristic environment created within a biofilm enhances both the proportion of hypermutable strains and the horizontal transfer of genetic material with consequent genetic rearrangement by recombination [30,31,32]. This makes *S. maltophilia* populations significantly complex and dynamic, being able to fluctuate rapidly under changing selective pressures.

Treatment of *S. maltophilia* infection is a challenge for clinicians due to its natural resistance to many antimicrobial drugs, mostly involving the study of strains isolated from CF patients [33,34]. Overall, our results confirmed the well-established multi-resistant phenotype of *S. maltophilia* in the hospital setting. Most strains, in fact, showed MDR phenotype (77.6%), with a frequency significantly higher in CF strains compared with non-CF strains (90% versus 66.7%, respectively).

SXT is the antibiotic of choice for treating *S. maltophilia* infections, although in recent years, increasing rates of resistance, ranging from 16% to 45%, were reported, especially in CF patients [9,35,36]. Considering available clinical breakpoints, our results confirmed SXT is the most active compound against all the strains tested. The overall resistance to SXT was 18.7%, although its activity was significantly higher in non-CF than CF strains (96.3% versus 75%, respectively), in agreement with previous findings [37,38].

Recently, a systematic review reported that fluoroquinolones show comparable effects on the mortality of *S. maltophilia* infection compared with trimethoprim-sulfamethoxazole, supporting the use of fluoroquinolones in clinical *S. maltophilia* infections [39], although high rates of resistance are also increasingly being reported. In the current study, the newer fluoroquinolone levofloxacin showed better activity against *S. maltophilia* than ciprofloxacin (susceptibility rates: 77.9% versus 18.8%, respectively), confirming previous studies [9,37,38].

We found that CF strains were significantly more resistant to piperacillin-tazobactam than non-CF strains (90% versus 53.3%, respectively). This finding might be the consequence of the airways’ concomitant colonization/infection by both *S. maltophilia* and *P. aeruginosa* observed in CF patients [35,40,41]. Consequently, the higher use of this antipseudomonal beta-lactam/beta-lactamase inhibitor combination in CF patients might have exerted a positive antibiotic pressure associated with higher levels of resistance.

To explore whether there was any correlation between biofilm formation and planktonic antibiotic resistance, first, we analyzed the biofilm biomass formed and the composition of the biofilm formation groups, with respect to resistance phenotypes. The most noteworthy information obtained from the present work, seen for the first time, was the overall negative relationship between the ability to form biofilm and the level of antibiotic resistance. A general trend was in fact seen; that is, the amount of biofilm formed was inversely correlated with the number of resistances to antibiotics. Confirming this trend, we observed that non-MDR strains were more efficient at forming biofilm compared to PDR strains; further, the frequency of non-producer strains among non-MDR strains was significantly higher compared with that shown by the multi-resistance phenotypes considered. We did not find any significant relationship between biofilm categories and resistance profiles, except for the CF population, where strains exhibiting more robust biofilm formation likely contained a larger proportion of MDR strains compared with strains from non-CF patients. This finding probably suggests that the relationship between drug susceptibility and biofilm formation is influenced by the environmental pressure at the site of infection.

Afterward, to determine whether biofilm formation is correlated with resistance to any antibiotic(s), we compared the biofilm forming capacities among strains with different resistance profiles to each of the antibiotics tested. A negative correlation between biofilm amount and resistance profile was observed in the case of piperacillin/tazobactam and meropenem, where the susceptible strains could form higher biofilm biomass amounts than resistant strains.

Susceptible bacteria reasonably need alternative strategies which are of no use to resistant bacteria, in order to escape antibiotic treatment and support their survival within the host. In agreement with earlier works [20,21,42], overall, our findings suggest that, also in *S. maltophilia*, susceptible bacteria may use biofilm formation in this regard, probably because the biofilm-mediated resistance might be less expensive in terms of energy requirements than chromosomal resistance mechanisms. However, the findings that nearly all strains unable to form biofilm maintain the MDR phenotype and show antibiotic-resistance rates comparable to those observed in biofilm-forming strains, indicate that other factors might be involved and should be examined to confirm such speculation.

## 4. Materials and Methods

### 4.1. Bacterial Strains and Growth Conditions

A total of 85 consecutive, non-repetitive *S. maltophilia* strains were isolated between 2017 and 2018: 40 from respiratory samples collected from CF patients attending the Cystic Fibrosis Unit at “Bambino Gesù” Hospital (Rome, Italy), and 45 from different sites (29 from respiratory tract, 11 from blood, three from anogenital swabs, and two from urine) of non-CF patients attended at various hospitals of central Italy.

All strains were grown at 37 °C onto Tryptone soy agar (Oxoid SpA; Rodano, Milan, Italy) and were identified as *S. maltophilia* using manual (API20NE) or automated (Vitek 2) systems (bio-Mérieux, Marcy l’Etoile, France). Strains were stored in Cryobank (Copan Diagnostics, Murrieta, CA, USA) at −80 °C and were cultivated in Tryptone soy broth (Oxoid SpA) at 37 °C for 18–20 h without shaking for further analysis.

### 4.2. Genetic Relatedness and Cluster Analysis

Bacterial DNA was digested with the restriction enzyme *XbaI* as previously described, with minor modifications [11]. PFGE was carried out using the following parameters: Initial switch and final switch times were 5 and 35 s, respectively; run time was 20 h, and the temperature was 12 °C for 20 h at 6.0 V/cm; and the included angle was 120°. Isolates with identical PFGE patterns were assigned to the same PFGE type and subtype. Isolates differing by one to three bands were considered genetically related and were assigned to the same PFGE type with different PFGE subtypes. Isolates with PFGE patterns differing by more than 4 bands were considered genetically unrelated and were assigned to different PFGE types. PFGE types were analyzed with BioNumerics software for Windows (version 2.5; Applied Maths, Ghent, Belgium). Normalization of DNA banding patterns was performed with bacteriophage lambda concatemer ladder standards. The banding patterns were compared by computer-assisted analysis using the unweighted pair group method with arithmetic averages (UPGMA) and with the Dice similarity coefficient. A tolerance of 1.5% in band position was applied during DNA pattern comparisons.

### 4.3. Antibiotic Susceptibility Testing

Six antibiotics were tested in vitro against *S. maltophilia* strains using the Kirby–Bauer disc diffusion technique (meropenem, ciprofloxacin, piperacillin/tazobactam, levofloxacin, and cotrimoxazole) or the broth microdilution method (chloramphenicol) according to the Clinical and Laboratory Standards Institute (CLSI) guidelines [22]. *Pseudomonas aeruginosa* ATCC 27853 was used as a reference strain. Strains categorized as resistant or intermediate, as defined using clinical breakpoints as interpretive criteria provided by CLSI [22], were grouped as “resistant” for data analysis. The activities of piperacillin-tazobactam, ciprofloxacin, and meropenem—whose CLSI breakpoints were not determined for *S. maltophilia*—were investigated using interpretation afforded for *P. aeruginosa*.

Since we tested the activity of antibiotics belonging to five different classes, according to Magiorakos et al. [24], we defined a strain as: Multidrug-resistant (MDR), if non-susceptible to at least one agent in three or more antimicrobial categories; extensively drug-resistant (XDR), if non-susceptible to at least one agent in four antimicrobial categories (i.e., bacterial isolates remain susceptible to only one category); pandrug-resistant (PDR), if non-susceptible to all agents in all antimicrobial categories (i.e., no agents tested as susceptible for that organism). Strains resistant to no more than 2 antimicrobial categories were described separately and referred to as non-MDR for the correlation analyses between antibiotic resistance and biofilm formation.

### 4.4. Biofilm Formation Assay

The ability of each isolate to form biofilm was quantitatively assessed using the microtiter plate method and crystal violet staining, as previously described [40]. In brief, overnight growth in TSB was adjusted to an optical density measured at 550 nm of 1.00 (corresponding to 1 × 10^9^ CFU/mL) and then diluted 1:100 in fresh TSB. Two-hundred microliters of this standardized inoculum was dispensed to each well of a sterile flat-bottom polystyrene 96-well microtiter tissue culture plate (Iwaki, Bibby srl; Milan, Italy) and incubation was at 37 °C, under an aerobic atmosphere. Control wells contained medium alone. After a 24 h incubation, non-adherent bacteria were removed by washing twice with 200 μL sterile phosphate-buffered saline pH 7.3 (PBS) (Sigma-Aldrich Italia, Milan, Italy), and biofilm amount was measured by crystal violet assay. Briefly, biofilm samples were fixed at 60 °C for 1 h, then stained for 5 min with 200 μL crystal violet. Excess stain was rinsed off with running tap water, and then the plates were air-dried. Crystal violet was extracted by exposure at room temperature for 15 min to 200 μL glacial acetic acid 33% (Sigma-Aldrich), and biofilm biomass (including adherent bacteria and EPS) was then assessed by measuring the optical density at 492 nm (OD_492_) (SpectraMax 190; Molecular Devices, Sunnyvale, CA, USA). Strains were classified into the following categories: No biofilm producer (OD_492_ ≤ ODc), weak biofilm producer (ODc < OD_492_ ≤ 2 × ODc), moderate biofilm producer (2 × ODc < OD_492_ ≤ 4 × ODc), and strong biofilm producer (4 × ODc < OD_492_), where ODc = mean OD_492_ of control (without inoculum) wells + 3 standard deviations (SDs) [23]. The reference *S. maltophilia* ATCC13637 strain was used as a positive control for strong biofilm formation (mean OD_492_: 2.629), whereas the *S. epidermidis* ATCC 12228 strain was used as negative control.

### 4.5. Statistical Analysis

Each experiment was carried out at least in triplicate and repeated on two different occasions (*n* ≥ 6). The Gaussian distribution of results was assessed by the D’Agostino and Pearson normality test. Differences in biofilm biomass between groups were evaluated by Mann–Whitney test, whereas differences between proportions were analyzed by Fisher’s exact test. Correlation between biofilm formation and antibiotic-resistance level was measured by linear regression analysis. All intermediate susceptibilities to the antibiotics were considered to be indicative of antibiotic resistance. Data analyses were carried out using GraphPad Software (Prism 7.0 for Windows; GraphPad software Inc.; San Diego, CA, USA), considering *p*-values less than 0.05 as statistically significant.

## 5. Conclusions

The findings from the present work indicated, for the first time in literature, that in *S. maltophilia* the possession of both classical drug resistance mechanisms in planktonic phase and the ability to form biofilm might contribute to the microorganism’s survival in a stressful environment, favoring its dissemination in the hospital setting.

The biofilm production probably acts as a survival mechanism for bacteria, especially in cases of susceptibility or when resistance level is not enough; bacteria that achieve resistance through biofilm production do not need to develop or maintain the mechanisms responsible for resistance of planktonic cells. Testing for biofilm formation is important in deciding the pathogenicity of clinical *S. maltophilia* isolates, and therefore, should be routinely performed in diagnostic laboratories. This evaluation might be paired with the antibiogram to predict possible cases of non-eradication of the pathogen and/or to apply synergic treatments facilitating antibiotic passage through the biofilm layer. However, considering the intrinsic antibiotic-resistance shown by sessile communities, our findings further indicate the poor therapeutic predictive value of the standard, planktonic-based, antibiogram and the consequent need to develop alternative, biofilm-based, antimicrobial susceptibility tests.

Our results also raise questions about the mechanisms underlying the complex relationship between biofilm formation ability and antibiotic resistance, and how resistant strains achieve high levels of biofilm-specific resistance despite producing lesser amounts of biofilms. Further studies are warranted to better clarify these mechanisms, in order to develop new and more effective prophylactic and therapeutic strategies for dealing with infections caused by *S. maltophilia*, an opportunistic and often multi-drug resistant, nosocomial pathogen.

## Figures and Tables

**Table 1:** Clonal relatedness, antibiotic-resistance, and biofilm formation of 85 *S. maltophilia* strains tested in the present study. Strains were genotyped by PFGE analysis. Susceptibility tests were performed using Kirby–Bauer disk diffusion agar (SXT, cotrimoxazole; LVX, levofloxacin; CPX, ciprofloxacin; TZP: Piperacillin/tazobactam; MER, meropenem) or broth microdilution technique (CHL, chloramphenicol) and interpreted according to CLSI guidelines [22]. Biofilm formation was assessed by spectrophotometric assay after crystal violet assay and the results were categorized according to Stepanovic et al. [23].

Strain ID	Isolation Site ^a^	PFGE Type	Susceptibility (S) or Resistance (R) to the Following Antibiotics:						Resistance Phenotype ^b^	Biofilm Formation	
			SXT	CHL	LVX	CPX	TZP	MER		Mean Value (OD_492_)	Class ^c^
SM31	sputum	1.1	S	R	S	S	R	R	MDR	<0.096	NP
SM10	sputum	2.1	S	R	S	R	R	R	XDR	0.633	S
SM172	sputum	3.1	S	R	S	R	R	R	XDR	0.432	S
SM176	sputum	3.1	S	R	S	R	S	R	MDR	0.615	S
SM186	blood	3.1	S	S	S	S	S	S	non-MDR	0.575	S
SM185	blood	3.1	S	S	R	R	R	R	MDR	2.282	S
SM143	sputum	3.1	S	R	S	R	R	R	XDR	2.750	S
SM27	sputum	3.1	S	R	S	R	R	R	XDR	0.455	S
SM173	sputum	4.1	S	S	S	R	R	R	MDR	0.574	S
SM139	CF	5.1	S	R	S	R	S	R	MDR	1.269	S
SM115	CF	6.1	S	S	S	R	R	R	MDR	0.329	M
SM119	CF	7.1	S	R	S	R	R	R	XDR	0.236	M
SM108	CF	8.1	R	S	S	R	R	R	XDR	0.165	M
SM190	CF	9.1	R	R	R	R	R	R	PDR	0.263	M
SM192	CF	9.1	R	R	R	R	R	R	PDR	0.446	S
SM193	CF	9.1	R	S	S	R	R	R	XDR	0.239	M
SM194	CF	9.1	S	S	R	R	R	R	MDR	0.184	W
SM195	CF	9.1	S	R	R	R	R	R	XDR	0.195	M
SM134	CF	9.1	S	S	R	R	R	R	XDR	0.547	S
SM135	CF	9.1	S	S	R	R	R	R	MDR	0.583	S
SM136	CF	9.1	R	R	R	R	R	R	PDR	0.541	S
SM106	CF	9.1	S	S	S	R	R	R	MDR	0.495	S
SM110	CF	9.1	R	R	R	R	R	R	PDR	0.475	S
SM171	sputum	10.1	S	S	S	R	R	S	non-MDR	1.099	S
SM123	CF	11.1	S	S	S	R	R	R	MDR	0.608	S
SM118	CF	12.1	S	S	S	R	S	R	non-MDR	0.755	S
SM116	CF	13.1	S	R	S	R	R	R	MDR	0.293	M
SM138	CF	14.1	S	R	S	R	R	R	XDR	0.391	S
SM109	CF	15.1	S	R	S	S	R	R	MDR	1.035	S
SM117	CF	16.1	S	R	S	R	R	R	XDR	<0.096	NP
SM113	CF	17.1	R	S	S	R	S	S	non-MDR	<0.096	NP
SM142	CF	18.1	S	R	S	R	R	R	XDR	1.545	S
SM47	sputum	19.1	S	R	S	S	S	R	non-MDR	0.100	W
SM157	CF	20.1	S	S	S	S	S	S	non-MDR	<0.096	NP
SM30	sputum	21.1	S	S	S	R	S	R	non-MDR	0.604	S
SM175	sputum	21.1	S	R	S	R	R	R	XDR	0.979	S
SM49	vaginal swab	22.1	S	R	S	S	S	S	non-MDR	0.746	S
SM130	CF	23.1	S	S	S	R	R	R	MDR	0.546	S
SM184	blood	24.1	S	R	S	R	R	R	XDR	<0.096	NP
SM42	pharynx swab	25.1	S	S	S	R	R	R	MDR	0.323	M
SM159	CF	26.1	R	S	S	S	R	R	MDR	0.370	M
SM4	urine	27.1	R	R	R	R	S	R	XDR	0.732	S
SM8	urine	27.1	R	S	R	R	S	R	MDR	1.767	S
SM24	sputum	27.1	R	S	R	R	S	R	MDR	0.629	S
SM37	blood	27.1	R	S	R	R	S	R	MDR	0.653	S
SM39	genital swab	27.1	R	R	R	R	R	R	PDR	0.459	S
SM51	blood	28.1	S	R	S	S	S	S	non-MDR	1.066	S
SM191	CF	29.1	S	R	S	R	R	R	XDR	0.337	M
SM144	CF	31.1	S	R	S	R	R	R	XDR	0.590	S
SM174	sputum	32.1	S	S	S	R	R	R	MDR	0.532	S
SM150	CF	33.1	S	S	S	R	R	R	MDR	0.391	S
SM180	blood	34.1	S	R	S	R	S	R	MDR	1.014	S
SM181	blood	35.1	S	R	S	R	R	S	MDR	<0.096	NP
SM137	CF	36.1	S	S	S	R	R	R	MDR	1.123	S
SM140	CF	37.1	S	R	S	R	R	R	XDR	0.233	M
SM32	sputum	38.1	S	S	S	S	S	S	non-MDR	0.509	S
SM21	rectal swab	39.1	S	R	R	R	R	R	XDR	0.320	M
SM36	sputum	40.1	S	S	S	R	S	S	non-MDR	3.646	S
SM48	blood	40.1	S	S	S	R	S	R	non-MDR	0.299	M
SM43	sputum	41.1	S	S	R	S	R	R	MDR	0.470	S
SM29	sputum	42.1	S	S	S	R	R	R	MDR	1.720	S
SM50	sputum	43.1	S	R	S	R	R	R	XDR	2.295	S
SM183	blood	44.1	S	S	S	R	R	R	MDR	0.397	S
SM107	CF	45.1	S	S	S	R	R	R	MDR	0.593	S
SM182	blood	46.1	S	S	S	R	S	R	non-MDR	0.831	S
SM104	CF	47.1	S	R	R	R	R	R	XDR	0.349	M
SM105	CF	48.1	R	R	R	R	R	R	PDR	0.581	S
SM120	CF	49.1	R	R	R	R	R	R	PDR	0.352	M
SM111	CF	50.1	S	S	R	R	R	R	MDR	<0.096	NP
SM112	CF	50.1	S	S	R	R	R	R	MDR	0.106	W
SM114	sputum	51.1	S	R	S	S	R	R	MDR	<0.096	NP
SM122	CF	52.1	S	S	S	R	R	R	MDR	1.300	S
SM45	sputum	53.1	S	S	S	R	S	R	non-MDR	0.596	S
SM170	sputum	53.2	S	R	R	R	R	R	XDR	0.521	S
SM46	blood	54.1	S	R	S	R	S	R	MDR	0.888	S
SM5	sputum	55.1	R	R	R	R	R	R	PDR	<0.096	NP
SM177	sputum	56.1	S	R	S	R	R	R	XDR	0.354	M
SM6	sputum	57.1	S	S	S	S	S	R	non-MDR	2.517	S
SM38	sputum	58.1	S	S	S	S	S	R	non-MDR	0.896	S
SM156	CF	59.1	S	S	S	R	R	R	MDR	0.276	M
SM7	pharynx swab	60.1	S	R	S	S	S	R	non-MDR	1.111	S
SM124	CF	61.1	S	S	S	S	R	R	non-MDR	<0.096	NP
SM14	sputum	62.1	S	S	S	S	S	S	non-MDR	0.847	S
SM40	pharynx swab	63.1	S	R	S	R	R	R	XDR	0.476	S
SM103	CF	64.1	S	S	S	R	R	R	MDR	0.400	S

^a^ Isolation site: CF, isolated from the airways of cystic fibrosis patients. ^b^ Resistance phenotype, according to Magiorakos et al. [24]: MDR, multidrug-resistant; XDR: Extensively drug-resistant; PDR, pandrug-resistant; non-MDR: Non multidrug-resistant. ^c^ Biofilm classes, according to Stepanovic et al. [23]: No biofilm producer (NP; OD_492_ ≤ OD_c_; OD_492_ ≤ 0.096), weak biofilm producer (W; OD_c_ < OD_492_ ≤ 2 × OD_c_; 0.096 < OD_492_ ≤ 0.192), moderate biofilm producer (M; 2 × OD_c_ < OD_492_ ≤ 4 × OD_c_; 0.192 < OD_492_ ≤ 0.384), and strong biofilm producer (S; 4 × OD_c_ < OD_492_; 0.384 < OD_492_), where OD_c_ = mean OD_492_ of control wells + 3 SDs.
